# Asafœtida a Preventive of Measles

**Published:** 1847-05

**Authors:** 


### ASAFŒTIDA A PREVENTIVE OF MEASLES.

Dr. Harper is not convinced by the arguments of Dr. Stahl, that asafœtida is not a preventive of measles. He writes us to say that his faith in the prophylactic is as great as ever, though he does not give any additional facts in support of his opinion.

